# European recommendations integrating genetic testing into multidisciplinary management of sudden cardiac death

**DOI:** 10.1038/s41431-019-0445-y

**Published:** 2019-06-24

**Authors:** Florence Fellmann, Carla G. van El, Philippe Charron, Katarzyna Michaud, Heidi C. Howard, Sarah N. Boers, Angus J. Clarke, Anne-Marie Duguet, Francesca Forzano, Silke Kauferstein, Hülya Kayserili, Anneke Lucassen, Álvaro Mendes, Christine Patch, Dragica Radojkovic, Emmanuelle Rial-Sebbag, Mary N. Sheppard, Anne-Marie Tassé, Sehime G. Temel, Antti Sajantila, Cristina Basso, Arthur A. M. Wilde, Martina C. Cornel, on behalf of European Society of Human Genetics, European Council of Legal Medicine, European Society of Cardiology working group on myocardial and pericardial diseases, European Reference Network for rare, low prevalence and complex diseases of the heart (ERN GUARD-Heart), Association for European Cardiovascular Pathology

**Affiliations:** 1 The ColLaboratory, University of Lausanne, Lausanne, Switzerland; 2 Section Community Genetics, Department of Clinical Genetics and Amsterdam Public Health research institute, Amsterdam UMC, Vrije Universiteit Amsterdam, Amsterdam, The Netherlands; 3 APHP, Referral center for inherited cardiac diseases, Sorbonne University, ICAN, INSERM UMRS1166, Hôpital Pitié-Salpêtrière, Paris, France; 4 European Reference Network for Rare and Low Prevalence Complex Diseases of the Heart (ERN GUARD-Heart), Amsterdam, The Netherlands; 5 University Center of Legal Medicine Lausanne-Geneva, Lausanne University Hospital and University of Lausanne, Lausanne, Switzerland; 6 Centre for Research Ethics and Bioethics, Uppsala University, Uppsala, Sweden; 7 Julius Center for Health Sciences and Primary Care, Department of Medical Humanities, University Medical Center Utrecht, Utrecht, The Netherlands; 8 Institute of Medical Genetics, Division of Cancer & Genetics, School of Medicine, Cardiff University, Cardiff, UK; 9 UMR 1027, Inserm, Université Paul Sabatier-Toulouse III, Toulouse Cedex, France; 10 Clinical Genetics Department, Guy’s & St Thomas’ NHS Foundation Trust, London, UK; 11 Institute of legal medicine, University of Frankfurt, Frankfurt, Germany; 12 Medical Genetics Department, Koç University School of Medicine (KUSoM), İstanbul, Turkey; 13 Clinical Ethics and Law, Faculty of Medicine, University of Southampton, Southampton, UK; 14 Clinical Genetics Service, University Hospitals Southampton NHS Foundation Trust, Southampton, UK; 15 UnIGENe and CGPP – Centre for Predictive and Preventive Genetics, IBMC – Institute for Molecular and Cell Biology, i3S – Instituto de Investigação e Inovação em Saúde, Universidade do Porto, Porto, Portugal; 16 Florence Nightingale Faculty, Nursing and Midwifery & Palliative Care, King’s College London, London, UK; 17 Genomics England, Queen Mary University of London, London, UK; 18 Institute of Molecular Genetics and Genetic Engineering (IMGGE), University of Belgrade, Belgrade, Serbia; 19 Cardiovascular Pathology, Molecular and Clinical Sciences Research Institute, St Georges Medical School, London, UK; 20 Public Population Project in Genomics and Society (P3G), McGill University and Genome Quebec Innovation Centre, Montreal, Canada; 21 Department of Medical Genetics and Department of Histology & Embryology, Faculty of Medicine, Bursa Uludag University, Gorukle, Bursa, Turkey; 22 Department of Forensic Medicine, University of Helsinki, Helsinki, Finland; 23 Cardiovascular Pathology Unit, Department of Cardiac, Thoracic and Vascular Sciences, University of Padua, Padua, Italy; 24 Amsterdam UMC, Heart Center; department of Clinical and Experimental Cardiology, Amsterdam Cardiovascular Sciences, University of Amsterdam, Amsterdam, The Netherlands

**Keywords:** Health policy, Genetic services

## Abstract

Sudden cardiac death (SCD) accounts for 10–20% of total mortality, i.e., one in five individuals will eventually die suddenly. Given the substantial genetic component of SCD in younger cases, postmortem genetic testing may be particularly useful in elucidating etiological factors in the cause of death in this subset. The identification of genes responsible for inherited cardiac diseases have led to the organization of cardiogenetic consultations in many countries worldwide. Expert recommendations are available, emphasizing the importance of genetic testing and appropriate information provision of affected individuals, as well as their relatives. However, the context of postmortem genetic testing raises some particular ethical, legal, and practical (including economic or financial) challenges. The Public and Professional Policy Committee of the European Society of Human Genetics (ESHG), together with international experts, developed recommendations on management of SCD after a workshop sponsored by the Brocher Foundation and ESHG in November 2016. These recommendations have been endorsed by the ESHG Board, the European Council of Legal Medicine, the European Society of Cardiology working group on myocardial and pericardial diseases, the ERN GUARD-HEART, and the Association for European Cardiovascular Pathology. They emphasize the importance of increasing the proportion of both medical and medicolegal autopsies and educating the professionals. Multidisciplinary collaboration is of utmost importance. Public funding should be allocated to reach these goals and allow public health evaluation.

## Background

After sudden unexpected death (SUD), forensic or clinical pathological examination may suggest an underlying cardiac disorder, which can be hereditary. These deaths can then be classified as cases of sudden cardiac death (SCD) [1, 2]. Taking personal and family history into consideration is of crucial importance and access to the related genetic information can be relevant for medical reasons (for example, to identify the possible cause(s) of death and then refine the prevention strategies for surviving relatives), as well as for public health or research purposes. Autopsy procedures are generally well described in various European regulations [3, 4], however, they often poorly incorporate postmortem genetic test information into the autopsy findings [5], and procedures differ between countries. The proportion of SUD in which autopsy takes place also varies among countries. This lack of connection between autopsies and genetic testing is highlighted by the increased potential of new technologies in genetics, to shed light on genetic mechanisms in SCD. At the same time, new techniques result in exponentially greater amounts of genetic data compared with former tests, much of which cannot yet be interpreted, or have uncertain significance. Distinguishing genetic results of clinical utility from the uncertain output needs expert interpretation and use of detailed phenotypic information. Moreover, conducting genetic or genomic testing in the context of postmortem DNA analysis raises practical, legal, and ethical challenges; including issues around consent, confidentiality and dissemination of familial information.

To address the lack of coordination between different professional domains and improve guidance on postmortem genetic testing for cardiac disorders, the Public and Professional Policy Committee of the European Society of Human Genetics (PPPC ESHG) organized a multidisciplinary Workshop sponsored by the Brocher Foundation and ESHG, on 23–25 November 2016. The workshop consisted of presentations by 12 experts in (forensic) pathology, cardiology, genetics, ethics and law, and group work to identify common challenges and draft recommendations. The workshop was attended by members of the PPPC, invited experts, and participants. After the workshop, a document listing recommendations was drafted, which was distributed among speakers and participants of the workshop to solicit comments. The recommendations were presented at several conferences in genetics and cardiology. A core writing group was formed and an updated draft was prepared for professional societies to discuss according to their own procedures for membership or expert consultation. The ESHG has posted this draft manuscript on its website for membership consultation from the end of March until April 30, 2018. After careful consideration of the suggestions, relevant comments were integrated, and in June–July 2018 the document was endorsed by the Board of the European Society of Human Genetics (ESHG), European Council of Legal Medicine (ECLM), European Society of Cardiology Working group on myocardial and pericardial diseases, Association for European Cardiovascular Pathology (AECVP), and European Reference Network for Rare and Low Prevalence Complex Diseases of the Heart (ERN GUARD-Heart).

## Introduction

Sudden cardiac death (SCD) is a major public health problem. Based on studies in the USA, the Netherlands, Ireland and China, SCD incidence ranges from 50 to 100 per 100,000 inhabitants annually and increases with age [6]. It accounts for 10–20% of total mortality, i.e., one in 5–10 individuals will eventually die suddenly [7]. Among these sudden death cases, the majority are SCDs [8]. For younger persons (under 40 years of age), the incidence of sudden death is lower, between 0.7 and 6.2/100,000 person-years [9, 10], and in ± 70% of cases the cause is cardiac [10, 11]. SCD can be caused by a number of underlying cardiovascular disorders. There are overall three categories: coronary artery disease, cardiomyopathies and no causative pathology (termed sudden arrhythmic death syndrome, SADS, if toxicology is performed, with no cause of death determined). Coronary artery disease is the most common cause of death in individuals over 35 years of age. Under the age of 35, SADS is the most frequent cause of death. In younger persons, genetically determined cardiac diseases (e.g., cardiomyopathies, ion-channel diseases) account for an important proportion of cases [1, 2, 10, 12–15]. Given the substantial genetic component of SCD in younger cases, postmortem genetic testing may be particularly useful in elucidating etiological factors in the cause of death in this subset [12].

It is well acknowledged that the results of such autopsies, including genetic testing, may be relevant for living blood relatives and public health prevention strategies.^1^ The identification of genes responsible for cardiac diseases, such as arrhythmic syndromes or cardiomyopathies, has led to the organization of cardiogenetic consultations in many countries worldwide. Expert recommendations are available, emphasizing the importance of genetic testing and appropriate information provision of affected individuals as well as their relatives [1, 13, 14, 16]. Furthermore, more research on this topic is recommended [17]. The goal of this paper is neither to review the specific clinical and pathological aspects of genetic cardiac diseases nor the diagnostic yield of genetic testing postmortem. However, the context of postmortem genetic testing raises some particular ethical, legal, and practical (including economic or financial) challenges, which are the subject of this paper.

An important challenge stems from the fact that general autopsy procedures are not always implemented and autopsy rates for SUD differ with age, region, and country [2, 10, 15]. A SUD most often will be investigated using forensic procedures, focused on ascertaining whether the cause of death is to be attributed to an underlying disease or if there is any legal implication, thereby distinguishing “natural” versus “unnatural” causes of SCD. Establishing the precise definition of the disease and informing the family members are not necessarily part of the aim of this procedure. There are also practical barriers related to organizational aspects, such as the lack of connection between the judicial system and the medical system. So far, it has been difficult to establish an effective communication between “postmortem professionals”, especially in the context of the forensic setting (pathologists), and specialized cardiogenetics experts. Insufficient communication between different medical specialties (i.e., pathology, cardiology, and genetics), further hinders the adequate provision of information to relatives of the deceased person [18]. Furthermore, there is a concern that medicolegal experts (forensic pathologists and/or medical examiners) may not have sufficient training and/or resources (including time) to properly interpret the genetic testing results [18, 19]. Clinical genetic services focused on a cautious approach, respectful of “the right-not-to-know”, when the clinical utility of genetic testing was low or absent [20, 21]. As genetic testing is increasingly able to identify conditions for which there is surveillance, prevention or treatment, a hypothetical right-not-to-know becomes more difficult to balance with a potential duty to warn [22–24]. Family members of an index patient diagnosed with, or suspected of, a heritable sudden cardiac death might not be aware of the death of their relative and/or of the possibility of an inherited genetic condition within the family, so that they might not have the opportunity to proactively look for appropriate advice and genetic information. A sudden cardiac death clearly prevents the seeking of consent for genetic testing and subsequent familial dissemination of relevant information, adding to clinical paralysis about what can legitimately be done with genetic findings in the deceased. Limited communication among family members and concerns about privacy might further complicate this. Furthermore, there is a lack of international guidance about the reporting of forensic postmortem genetic test results with few local practice guidelines [18, 19, 25].

**Fig. 1:**
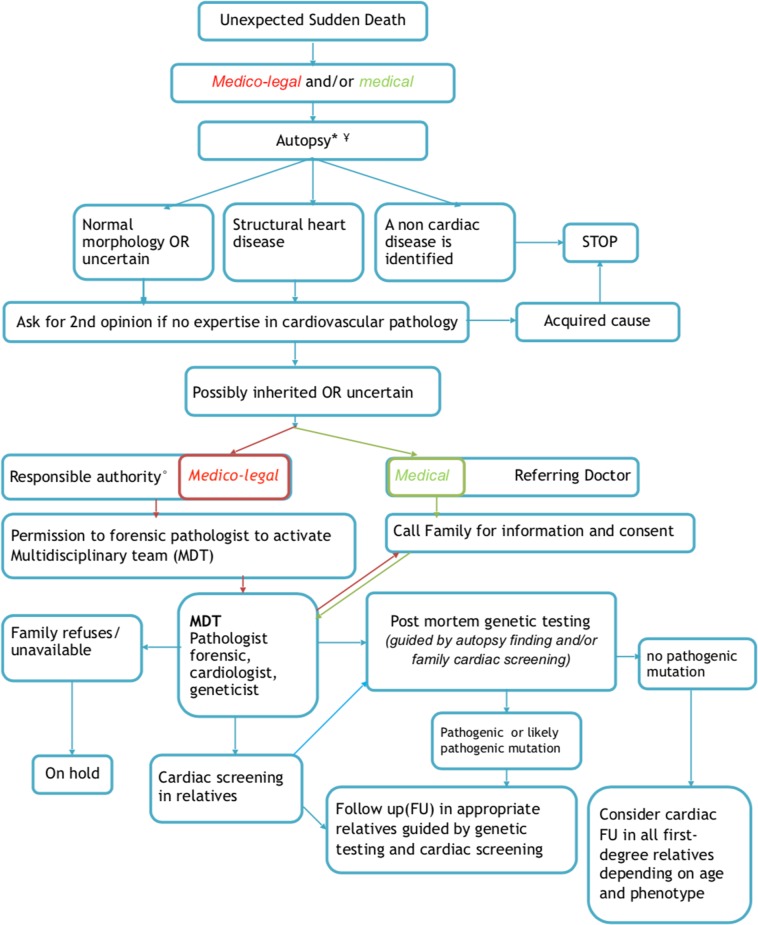
Flowchart. MDT multidisciplinary team. *Mandatory if <40 y; consider if >40 and <65y; Case by case >65 y; ^¥^Standards: minimal criteria; histological examination; sampling for toxicology, genetics, other lab tests; collection of health history/records. °Depending on the country (judge, coroner,…)

In the following paragraphs, we will summarize specific procedural, ethical, legal, and practical challenges for post-mortem genetic testing after sudden cardiac death that have been discussed during the workshop. The key elements that would ideally need to be addressed will be depicted in the flowchart (Fig. 1), though the actual organization of these actions may vary between countries or jurisdictions. We will conclude by making recommendations on how best to include postmortem genetic testing in the context of SCD in order to contribute to the identification of the cause of death, and then contribute to a better management of relatives by optimizing screening strategies and the treatment of preventable disorders.

## Sudden cardiac death

In this document, we will focus on cardiovascular disorders. Sudden death (SD) “is a non-traumatic, unexpected fatal event occurring within 1 h of the onset of symptoms in an apparently healthy subject. If death is not witnessed, the definition applies when the victim was in good health 24 h before the event” [1]. The term sudden cardiac death (SCD) “is used when a congenital, or acquired, potentially fatal cardiac condition was known to be present during life; OR autopsy has identified a cardiac or vascular anomaly as the probable cause of the event; OR no obvious extra-cardiac causes have been identified by postmortem examination and therefore an arrhythmic event is a likely cause of death” [1]. Knowing the cause of sudden death may be particularly relevant for family members, (i) if the condition is hereditary and therefore relatives might also be at risk, and (ii) if prevention or treatment is available. Apparently healthy people who die while performing sports, during sleep, while swimming, or while driving may be victims of arrhythmias as a consequence of a cardiogenetic condition. Some cardiogenetic conditions might be identified in the course of the autopsy, e.g., in case of myocardial disease, such as cardiomyopathies. However, a number of cases remain unexplained after complete autopsy including laboratory analyzes, referred to as sudden arrhythmic death syndrome (SADS), in which the underlying mechanism of death might be an arrhythmia due to an inherited ion-channel disorder [2, 13]. Our focus will be on individuals over 1-year old, since implications related to sudden infant death syndrome (SIDS) are somewhat different and protocols for management—including information for the family—are more clearly defined. SIDS is defined as the unexpected death of a seemingly healthy infant less than a year old with no cause of death determined after a full medicolegal investigation [1]. We, however, acknowledge presumed SIDS cases might be related to a genetic cause in up to 20% of cases (related to inherited cardiac diseases, such as channelopathies or cardiomyopathies) [26], but the proportion of explained cases was less than 5% in recent studies [27], and alternative non-cardiac monogenic causes may also occasionally be responsible [28].

### Postmortem investigations in different countries

How postmortem investigations are organized differs between countries. For forensic purposes, the procedures in place reflect the aim of investigating the potential involvement of third parties (e.g., homicide or an accident) or self-inflicted death, while for the medical context a different system exists that aims to discover the underlying pathological cause of unexpected death [2]. The first is often called “medicolegal autopsy”, the second “medical autopsy”. For some of these contexts and in some countries, autopsy may be mandated (e.g., in sudden infant death), while for other situations relatives may be asked for permission to perform autopsy, but this is not consistent across Europe. Health insurance may end with a person’s death, so funding for an autopsy and/or genetic testing may be a major obstacle even when it may be of benefit to surviving relatives.

### Aim of a medicolegal autopsy

The traditional aim of a medicolegal autopsy is to ascertain the cause of death in cases of unexpected, sudden death. If homicide, suicide, or an accident can be ruled out, then a “natural death” is assumed. Especially in younger cases, “natural causes” may be related to underlying genetic conditions, such as a cardiogenetic disorder. Given the potential medical relevance of autopsy information for family members, autopsies, including medicolegal autopsies, should have a dual aim of both ascertaining the cause of death and providing information to family members in case the findings indicate a substantial risk that family members may also develop the disease.

### Percentage of autopsies performed

There is considerable variation among countries and regions in the numbers of autopsies conducted and for many countries data are missing. In a study from the Netherlands, an autopsy was found to be performed in about 43% of sudden deaths of persons aged 1–44 years, in Denmark this has been found to be 70% 1–35 and 60% 1–49 [10, 15, 29]. In many countries, the rate is unknown. In the UK, in all cases of sudden unexpected death a coroner is involved and an autopsy is required. In some other countries medical professionals, such as general practitioners may be primarily responsible for establishing the cause of death and varied arrangements may exist in different jurisdictions for requesting an autopsy, whether medical or medicolegal. Even though in 1999, the Committee of Ministers of the European Council adopted a recommendation on the harmonization of medicolegal autopsy rules and established clear criteria in what circumstances an autopsy is required [3, 4, 30], including sudden, unexpected death, there is an urgent need to instruct the relevant professionals regarding these criteria [2].

### Need for a full autopsy

A full autopsy (i.e., including dissection of internal organs in all body cavities, macroscopic and histologic examination of those organs, and utilizing modern postmortem laboratory (toxicology, biochemistry, and microbiology) tests, and storage of adequate samples for genetic testing) is necessary to identify cases where a cardiac disorder is the likely cause of death and to collect supporting evidence. Procedural and technical guidance is available in a published protocol [2], which should be implemented in all European countries. This protocol provided by the Association for European Cardiovascular Pathology (AECVP) includes a complete analysis of the heart. Funding may be a major obstacle in increasing the number of autopsies where postmortem investigation is not mandatory. In the UK, for instance, all full autopsies are funded by the coroner, but specialist cardiac examination is funded by the charity CRY(Cardiac Risk in the Young; http://www.c-r-y.org.uk/). The need for such an expert cardiac pathological opinion is apparent, as referring pathologists tend to overcall structural disease and underdiagnose SADS [31], while uncertain findings at autopsy may still harbor genetic etiologies [32]. Moreover, the absence of a common protocol of investigation and shared diagnostic criteria at postmortem are main causes of the nonuniform reporting of the causes of death among different series, and a clear example is atherosclerotic coronary artery disease, which is underrepresented in many studies [2]. To allow for sufficient expertise and standard procedures, regional centers and/or experts in examination of the heart would be ideal, as would appropriate funding for the necessary investigations.

### Genetic testing in relation to full autopsy

In many European countries, legal provisions do not allow pathologists to request genetic testing after a full autopsy including a thorough examination of the heart, and only geneticists can order a genetic test. In the practice of forensic autopsies, a genetic test can be requested for diagnostic purposes in some countries. However, the interpretation of genetic test results for inherited cardiac arrhythmia disorders requires highly specialized expertise [33] and it may be difficult, if not impossible, to use the test as a diagnostic tool. If there is a clear indication for genetic testing, for instance, in case of inherited cardiac disease, such as cardiomyopathy, a panel of genes related to the condition could be used. However, the indication for genetic testing is more debated when the phenotype is unclear (the autopsy is normal, unexplained SCD, SADS). Clear pathogenic results may offer immediate benefit to families [34], but these form a minority of cases. Variants of uncertain significance (VUS) will be found in many cases, and interpretation of the results would then require not only phenotypic information of the deceased but also family investigation to see whether the genotype segregates with a cardiac phenotype. If variants are proven as de novo, then this increases the potential utility. Otherwise, more extensive testing is necessary. Often, at most an indication of the possible diagnosis may be obtained but without confirmation. However, it has recently been shown that combining postmortem testing and family investigation can lead to a diagnostic yield of 40% [34, 35]. In practice, it may take too long for the test results to be available for them to be included in the final autopsy report. Moreover, genetic analysis currently remains expensive despite advances in technology, and coroners may be reluctant to pay for investigations that have no impact on the judicial procedure. To allow for future genetic analysis, in the course of an autopsy, a blood or tissue sample (spleen, muscle, skin, and kidney) should be taken and stored frozen together with detailed phenotypic information.

### Storing samples for future genetic testing

If a DNA sample (or fresh frozen tissue from which DNA could be extracted) is stored, it becomes possible to use the sample for testing in the future on family request, and potentially for research. Consent for such DNA testing and storage in clinical or biobank repositories is not a trivial matter. Guidelines and legislation about who can or should give such consent varies between countries. In the UK for example, the Human Tissue Act recognizes that the spouse of a deceased person—although often the person with whom consent is discussed—does not have the same interests in such storage as biologically related family members. Refusal of consent for storage by a spouse could deny blood relatives important information, and so should only be accepted in case of an informed refusal and if there is no one else who can provide relevant consent.

Sample storage requires stringent and good communication between the forensic pathologist and relevant clinicians (e.g., geneticists or the cardiogenetic department). Sample handling and storage should be part of standard procedures. Currently, European recommendations exist about the use of samples from a deceased person. In 2004, a multidisciplinary expert group invited by the European Commission published 25 recommendations on the ethical, legal, and social implications of genetic testing [36]. The 24th recommendation addresses postmortem genetic analysis. It was stated that member states are to take action to promote the right of access to samples and data from a deceased person, in the case of the overriding interest of blood relatives. However, in practice, forensic departments often do not have the facilities to store material for a long period of time. Long-term storage in clinical or biobanking systems, and subsequent access by family members, raise questions about what type of consent is appropriate.

### Informing the family

After medicolegal autopsy, a report is sent to the representative of the legal system (e.g., coroner, district attorney, or police). However, whether and how the family is involved in receiving the results of the autopsy differs between countries. Even if information is provided to family members about an underlying hereditary cardiovascular disorder, it may not be clear to them whether this might be relevant for their own health or how they can act on the information by seeking appropriate referral. More work to close the gap between medicolegal reporting about the deceased and the appropriate care of relatives is required. Examples include: establishing procedures for providing “family letters” that provide clear information on the possibility of a genetic disorder and where further information and care can be obtained from; making clear who is responsible for providing such information or letters; having accessible online information and/or dedicated medical professionals that can be contacted by families or by their general practitioners. These procedures, however, do not ensure that all relevant relatives will be reached. Intrafamilial communication of genetic risk information is a complex, multifaceted process, and ongoing support for the communication with family members is often needed.

Family members also need to be informed when a medical autopsy is performed if the results indicate the possibility of an underlying hereditary cardiovascular problem. This requires good communication and collaboration between, most notably, the pathologist, the physician, and/or coroner who ordered the postmortem investigation, the genetics department and the general practitioner (family doctor).

### Consent

In the context of a forensic investigation, explicit consent is usually not required for postmortem investigations. It is common practice to obtain consent from a patient to contact family members during life, but this is obviously not possible after the patient’s death. Since relatives may have an interest in knowing about the deceased’s results, and serious harm might be prevented through their gaining this knowledge, this is generally held to tip the balance in favor of familial disclosure as opposed to any professional obligation to maintain confidentiality after death. However, ethical debate continues [22, 23]. The communication of relevant information is difficult to standardize, but needs careful balancing of relevant information in a clear manner that allows relatives to make informed decisions about whether or not they want to pursue investigations. Furthermore deciding who is informed and when is not a trivial task. Clinical Genetics Services have several tools in their armoury to facilitate such familial communication, so early contact or referral is recommended where possible.

### Family investigation

The first step in family investigation is a cardiology referral of first-degree family members, either in case of a clear autopsy diagnosis of a cardiac disease that is usually inherited, or in case of no clear cardiac disease diagnosed at autopsy. The genetic investigations should be performed in the first instance on the deceased person’s sample as a general requirement before any predictive testing in apparently healthy relatives. The genetic analyzes are based on a targeted gene panel when a clear cardiac disease has been identified at autopsy, or might be enlarged to large gene panels or even exome sequencing in case of SADS [34, 37, 38]. However, in the last situation (when no clear cardiac disease is identified), the interpretation of genetic results should be conducted with great caution and can be considered as hypothesis generating rather than establishing the cause of death. Predictive genetic testing of relatives can then be offered in cases where a variant affecting function (or pathogenic variant according to the ACMG 2015 guidelines [39]) has been detected in the deceased and a reasonable link with the death is made. When there are no genetic test results in the deceased person but a cardiac disease is identified in a relative after systematic screening, then genetic testing can be performed in this particular relative. A careful consideration of clinical information and investigations on the deceased and on the relatives will be essential for a meaningful interpretation of the genetic test results; it will often be wise for this to be conducted as a multidisciplinary process.

### Multidisciplinary collaboration

To connect the hitherto distinct fields of forensics, pathology, genetics (clinical and laboratory, including bioinformatics expertise), and cardiology, the establishment of multidisciplinary teams is vital. In many places, current collaborations between cardiologists and geneticists can be the basis of such teams. These multidisciplinary teams should ideally include adult and pediatric cardiologists, geneticists, legal and medical pathologists, and psychologists. Their roles are (i) to examine individual cases to improve their management and assist in evaluating and reevaluating whether the found genetic variants (pathogenic or likely pathogenic variant) should be used for investigation in the families; (ii) to share information on management of samples, tests results, and family investigations so as to enhance the overall management of each case as well as to improve the practice and expertise of individual professionals; (iii) to support professionals seeking advice; (iv) to designate a case manager that can be contacted by healthcare and forensic professionals as point of reference; (v) to contribute to establishing local and national protocols and improve their implementation; (vi) to collaborate with the relevant institutions to collect and provide information about critical or strategic matters for public health purposes.

Professionals and policy makers are encouraged to distribute and discuss the following recommendations to improve practices relating to postmortem genetic testing for cardiac disorders, and to stimulate the development of national and European guidance. This task requires the sustained and collaborative effort by the various professional groups involved.

## Recommendations

1. Sudden cardiac death at a young age should be considered as a public health priority because of the high prevalence of inherited cardiac diseases and the impact for the family. Therefore, public funding should be allocated for related relevant investigations.
2. Increasing the proportion of both medicolegal and medical autopsy in case of sudden, unexpected natural death should be a major objective. This should be mandatory for deaths under the age of 40, it should be considered for deaths between ages 40 and 65, and evaluated on a case by case basis after age 65.
3. Educate primary care physicians, coroners/district attorneys, and (forensic) pathologists on when an autopsy should be performed.
4. Medicolegal autopsies should have a dual aim: (i) to establish if a death was natural or caused by a criminal act or accident; (ii) to establish the cause of a natural death and allow results to be used for preventive healthcare for the surviving relatives.
5. In cases of sudden (cardiac) death, a full autopsy should be performed, including heart dissection, sampling for possible genetic and toxicological analysis, and examination should adhere to minimal standards as per European guidelines. Guidelines should be made mandatory in European countries by seeking support from Ministries of Health and Justice.
6. Access to second opinions of teams with expertise in cardiovascular pathology (reference network) should be promoted to support routine workup.
7. In the course of an autopsy, blood or tissue samples (e.g., spleen, muscle, skin, and kidney) should be taken and stored frozen together with detailed phenotypic information for future genetic analysis in the setting of a suspected inherited disorder or normal/equivocal cardiac autopsy. This sample should be accessible for medical purposes. After completion of the forensic proceeding, the sample should be stored in healthcare-embedded biobanks according to national regulations. Family members should be informed about the availability of the sample and asked for consent to storage. It should be clear how long a sample will be stored.
8. Organize multidisciplinary teams or reference centers to connect different domains in healthcare and the judiciary system in order to co-design pathways and procedures, clarify who is responsible for storage during and after the forensic investigation, clarify the source of funding to implement this policy, design information letters or leaflets for patients and family members, and designate the case manager who can be contacted by healthcare and forensic professionals as a point of reference.
9. Information on genetic testing and communication of genetic test results should be given in compliance with standard procedures in clinical genetics and with the appropriate national legislation. Familial communication and appropriate cascade testing should be approached in a systematic fashion using genetic services where possible. We consider that there can be no duty to warn all relatives, but that a responsible system will make attempts to alert relatives when appropriate.
10. A multidisciplinary cardiogenetic team should conduct the family investigation. The appropriate genetic test should be considered according to a combination of pathology findings, family history, and the results of cardiac family screening. The genetic test should be performed on the DNA of the deceased in the first instance, and testing of relatives should then be offered if a variant affecting function (pathogenic or likely pathogenic variant) is identified.
11. Professionals, professional organizations, relevant national institutions, and policy makers should make a collaborative effort to further discuss the respective responsibilities of the different professionals involved, the allocation of funding for autopsies and postmortem genetic tests, the procedures required to connect the domains of forensics and healthcare in the context of hereditary cardiac disorders identified in suddenly deceased individuals, and how best to address the ethical issues arising when informing family members and possible psychological harms associated with disclosure.
12. There is a need for economic evidence and public health evaluation to identify the incremental costs and consequences of the use of genetic testing in postmortem investigations compared with current practice to clarify the situations for which postmortem genetic testing is an effective use of finite budgets.

## Footnotes

1 In this document we will concentrate on cardiovascular disorders. The resulting guidance may inspire efforts to integrate postmortem genetic testing for other purposes. For instance, genetic susceptibility to adverse drug reactions could be relevant both for forensic and public health purposes.
